# Fatty infiltration of the cervical multifidus musculature and its clinical correlation to cervical spondylosis

**DOI:** 10.1186/s12891-023-06595-4

**Published:** 2023-07-27

**Authors:** Zhifei Li, Qinqiu Liang, He Li, Xiaocheng Lin, Jiwen Meng, Daishui Yang, Chengwei Li, Yuanyao Liang, Yin Yang, Yuanfang Lin, Ziyang Liang

**Affiliations:** 1 Department of Spine Surgery, The First Affiliated Hospital of Guangxi University of Chinese medicine, Nanning, 530023 Guangxi China; 2 Guangxi University of Chinese medicine, Nanning, 530001 Guangxi China; 3 Department of Spine Manipulation, Shenzhen Traditional Chinese Medicine Hospital, The Fourth Clinical Medical College of Guangzhou University of Chinese Medicine, Shenzhen, 518033 Guangdong China; 4 Macalester College, Saint Paul, MN 55105 USA; 5 Department of Orthopaedic, Second Xiangya Hospital of Central South University, Changsha, 410011 Hunan China

**Keywords:** Fat infiltration, Cervical multifidus, Cross-sectional area, Radiological measurement, Correlation analysis

## Abstract

**Purpose:**

Fat infiltration (FI) of the deep neck extensor muscles has been shown to be associated with poor outcomes in cervical injury, mechanical neck pain, and axial symptoms after cervical spine surgery. However, information is scarce on the severity of FI in cervical extensors associated with different clinical syndromes in patients with cervical spondylosis.

**Objective:**

To investigate the relationship between the severity of FI in the cervical multifidus musculature and its clinical correlates in the syndromes and sagittal alignment of patients with cervical spondylosis.

**Methods:**

This study was conducted as a retrospective study of twenty-eight healthy volunteers (HV) together with sixty-six patients who underwent cervical radiculopathy (CR), degenerative myelopathy (DM), and axial joint pain (AJP) from January 2020 to March 2022. MRI was used to measure the fat cross-sectional area (FCSA), functional muscle cross-sectional area (FMCSA), total muscle cross-sectional area (TMCSA), FI ratio of the cervical multifidus musculature at each cervical level from the C3 to C6 segments and the cervical lordosis angle in the included subjects.

**Results:**

The difference in the FCSA and FI ratio in patient groups with cervical spondylosis was significantly greater than that of the HV group (*P* < 0.05), and the Cobb angle of the DM group, AJP group and HV group was significantly greater than that of the CR group (*P* < 0.05). The FI ratio comparison showed no significant difference by sex, and the comparison of FCSA, FMCSA, TMCSA and FI ratio showed no significant difference by age range from 35 to 69 in the included subjects. The FCSA and TMCSA in patients with cervical spondylosis were positively related to the Cobb angle (r_s_= 0.336, *P* = 0.006, r_s_ =0.319, *P* = 0.009, respectively), and the FI ratio was inversely correlated with the Cobb angle (r_s_= -0.285, *P* = 0.020) and positively correlated with age (r_s_ =0.261, *P* = 0.034). In the HV group, FMCSA was inversely correlated with age (r_s_= -0.400, *P* = 0.035), while the FI ratio had a positive correlation with age (r_s_= -0.423, *P* = 0.025).

**Conclusion:**

Compared with healthy subjects, a more severe degree of FI in the multifidus musculature and sagittal imbalance were found in patients with cervical spondylosis. These two imaging features are considered to be important concomitant phenomena of cervical spondylosis, and the more severe FI is, the worse the sagittal imbalance. However, each syndrome had no obvious difference in FI in the multifidus musculature.

## Introduction

Fat infiltration (FI) is the pathological infiltration of skeletal muscle [1, 2]. FI of the deep extensor paraspinal muscles has been shown to be associated with poor outcomes in cervical injury [3, 4], mechanical neck pain [5, 6], and axial symptoms after cervical spine surgery [7]. This may occur because the deep neck extensor muscles, including the primary multifidus musculature, play a critical role in postural biomechanics through their deep attachments to the cervical spine [8–10]. They reinforce lordosis during rotation and antagonize flexion of the cervical spine [11]. There remain several mechanistic pathways for the occurrence of FI including, but not limited to ageing, disuses, direct injury, and denervation of skeletal muscle [12–14]. Current studies have focused mainly on the relationship between FI in the cervical multifidus and mechanical neck pain, including those with whiplash-associated disorders and chronic idiopathic neck pain [15, 16]. The loss of muscle strength and spinal stability due to FI could increase the risk of mechanical neck pain [17]. However, several studies have also demonstrated that increased FI is associated with degenerative osteoarthritis of the spine, which could contribute to a decrease in spinal joint mobility [6, 18]. Hence, FI in the cervical extensor muscles is probably an important feature of cervical spondylosis.

Cervical spondylosis is a general and nonspecific term that encompasses a broad spectrum of afflictions but, for purposes of clarity, can be organized into three clinical syndromes: Type I Syndrome (Cervical Radiculopathy, CR); Type II Syndrome (Cervical Myelopathy, CM); and Type III Syndrome (Axial Joint Pain, AJP) [19]. The first two reflect neurologic involvement, whereas the third represents painful joint dysfunction. To date, the study is still in its infancy on the severity of FI in cervical extensors associated with different clinical syndromes in patients with cervical spondylosis. FI induced muscle denervation may develop in the spinal cord and nerve root compression in patients with cervical spondylosis. FI of cervical spinal musculature is emerging as a potentially propulsive factor of disability [20]. An improved understanding of specific pathophysiological processes may inform both the clinical assessment of and management for patients with different clinical syndromes in patients with cervical spondylosis.

Therefore, the purpose of this preliminary study is to examine whether patients with different clinical syndromes of cervical spondylosis demonstrate FI in the deep muscles of the cervical spine when compared to age-matched controls. The weakness of cervical extensor muscles causes loss of cervical lordosis [21], and FI is the pathological infiltration of muscle. We also questioned the influence of FI in the cervical extensor muscle on the loss of cervical lordosis. MRI was used to quantify the pathobiology of changes in atrophy and FI of the cervical multifidus musculature in this study, as it presents the best and most precise noninvasive examination of muscle morphometry and composition [22].

## Materials and methods

### Participants

All subjects consented to inclusion in the study, and this study was conducted after obtaining institutional review board approval from our institution (IRB number 2022JJ40696). The records of patients from January 2020 to March 2022 were retrospectively reviewed. We enrolled a cohort of sixty-six patients with cervical spondylosis and twenty-eight age-matched, asymptomatic controls to undergo imaging of the cervical spine for analysis of multifidus FI. All patients with cervical spondylosis were diagnosed and organized into three clinical syndromes: CR syndrome (Twenty-one subjects), CM syndrome (Twenty-one subjects) and AJP syndrome (Twenty-four subjects) [19] based on both clinical and radiographic findings: ①Inclusion criteria for the following in CR syndrome: classic symptoms as neck pain with radiating upper extremity pain and/or weakness and/or numbness AND exam findings of the Spurling sign (+) AND radiographic signs of nerve root compression. ②Inclusion criteria for the following in CM syndrome: classic symptoms as weakness of all four extremities together with a sensory level below which there is reduced or absent appreciation of pain, touch, vibration, or position sense AND exam findings of weakness, hyperreflexia, or change in coordination AND radiographic signs of spinal compression. ③Inclusion criteria for the following in AJP syndrome: classic symptoms as neck pain together with radiation to one or more of the following: the medial scapula, chest wall, shoulder area, and head AND exam finding of no neurologic deficit AND movement of the neck probably produces pain. Exclusion criteria included the following: age < 35 or > 70, comorbid neural disease (e.g., multiple sclerosis), pregnancy or nursing, active systemic rheumatological disease, active peripheral or vascular neuropathy, and urgent need for surgery. In addition to the exclusion criteria for patients, controls were also screened for neck pain, or history of spinal surgery.

### MRI measures and analysis

**Fig. 1:**
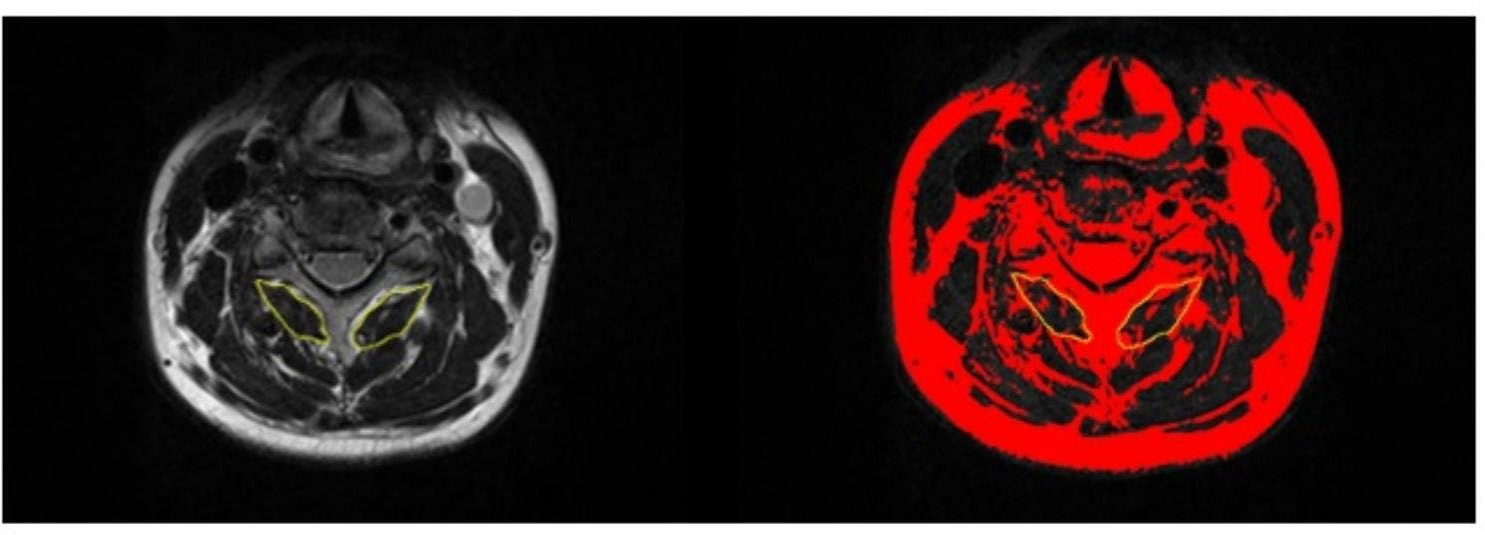
TMCSA and FI ratio of the multifidus muscle were measured. (Left) This area within the yellow line represents the multifidus TMCSA. (Right) The red area within the yellow line represents the fatty amount of the multifidus

Imaging data of all subjects were collected with a 3.0T MRI scanner (Siemens, Erlangen, Germany). Each participant underwent magnetic resonance examination of the cervical spine, which included obtaining a localizer scan and performing a T2-weighted sagittal turbo spin echo sequence to determine the location of the fat-water scan. A standard 12-channel head coil and a 4-channel neck coil were used as receiver coils to improve signal-to-noise. The axial FLASH dual echo, gradient echo sequence had a duration of 4:23 min, an in-plane resolution of 1.0 mm using a rectangular field of view of 75%, and a thickness of 3 mm with slab oversampling of 22% and 36 partitions to prevent aliasing in the 3D (superior-inferior) direction. The TR/TE1/TE2 6.59/2.45/3.68 ms with a field of view of 224 × 320 mm, and covered the cephalad portion of C3 through the caudal portion of the C7 vertebral endplate [20]. The subjects were positioned supine on the scanning bed so that the audionasal line was perpendicular to the scanning neutral position of the bed. The axial plane of the C3—C6 intervertebral discs and the median sagittal plane of the cervical spine were selected from T2-weighted images (T2WI), which were measured and analysed by ImageJ software (version 1.52, NIH, USA). As shown in Fig. 1, the desired muscle area was initially mapped along the muscle edges of the multifidus muscle, and the total muscle cross-sectional area (TMCSA) was measured. Pseudocolouring techniques were employed to dye bright pixels of adipose tissue red, and the area of the red region within the muscle, referred to as the fat cross-sectional area (FCSA), was then calculated based on image proportions. The functional muscle cross-sectional area (FMCSA) was calculated by subtracting FCSA from TMCSA (TMCSA minus FCSA). The degree of FI was expressed as the FI ratio and calculated by dividing FCSA by TMCSA. All data from the MRI image measurement were averaged by three orthopaedic surgeons.

**Fig. 2:**
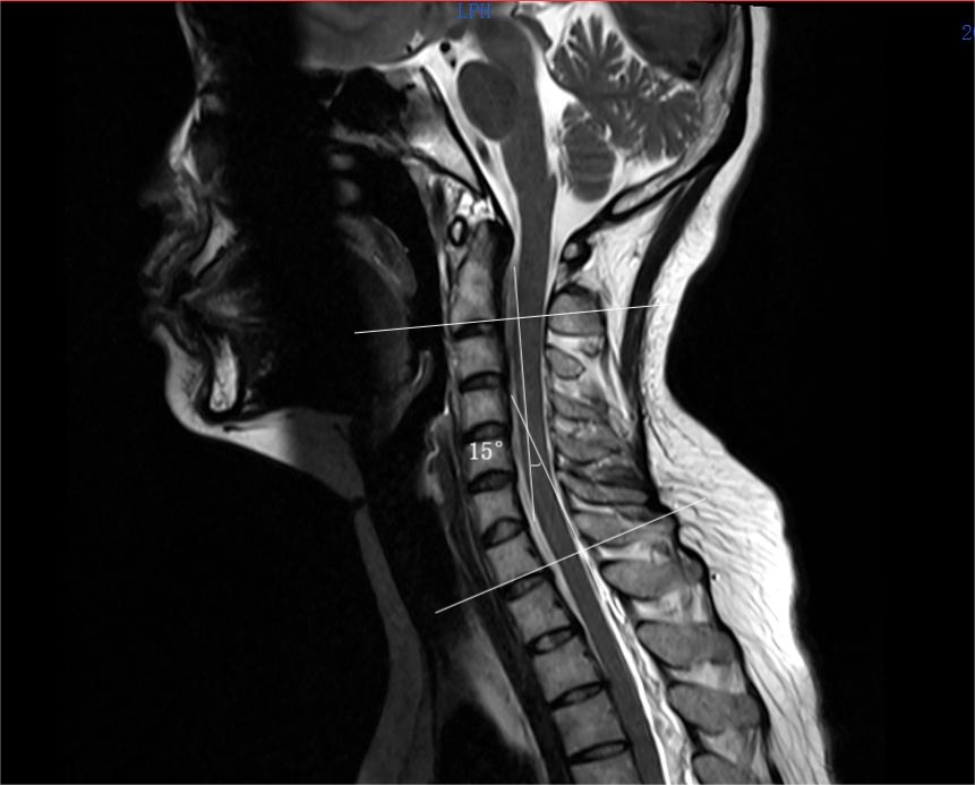
Depiction of the measurement of the C2-C7 Cobb angle. The C2-C7 Cobb angle is defined as the angle between two lines perpendicular to the lower endplates of C2 and C7

In Fig. 2, the cervical lordosis angle was measured using the C2-C7 Cobb angle. To measure the angle, a horizontal line was drawn along the lower endplates of C2 and C7 on a median sagittal plane (T2WI) using the Picture Archiving and Communication System. Then, a vertical line was drawn along each of the two horizontal lines, and the intersection of the two vertical lines was defined as the Cobb angle.

### Statistical analysis

The data obtained were analysed using SPSS 26.0 statistical software. Descriptive analysis and normality tests were performed on all data. Continuous variables with a normal distribution are presented as $x\pm s$, while those with a nonnormal distribution are presented as quartiles, since they are not affected by the maximum or minimum value. Categorical variables are presented as enumeration data. Differences between the study group and control group were examined using univariate analysis of variance, and the independent sample t test was used to compare differences between genders. Pearson and Spearman correlation analyses were used to investigate the correlation of age, BMI, and Cobb angle with multifidus FI ratio, FMCSA, and TMCSA, with *P*<0.05 indicating statistically significant differences.

## Results

The demographic characteristics of the four groups are presented in Table 1. There were no significant differences between groups in gender, age, and BMI (Gender; *F* = 2.532, *P* = 0.470, age; *H* = 3.294, *P* = 0.349, BMI; *F* = 0.496, *P* = 0.686). Patients with cervical spondylosis and controls were similar with respect to most baseline demographic and clinical characteristics.

**Table 1:** Patient Characteristics

Descriptive	AJP group (n = 24)	CR group (n = 21)	CM group (n = 21)	HV group (n = 28)	*F/H* value	*P* value
Gender (M/F)	12/12	9/12	14/7	15/13	2.532	Ns
Age (years)	54.50 (48.25, 58.00)	53.00 (44.00, 59.00)	52.00 (48.50, 68.00)	52.50 (44.25, 56.75)	3.294	Ns
BMI (kg/m^2^)	23.79 ± 3.06	24.40 ± 3.62	23.28 ± 2.93	23.73 ± 2.41	0.496	Ns

Ns, nonsignificant

Of the patients with cervical spondylosis, the FCSA and FI ratio were significantly higher than in the HV group in Table 2 (FCSA; *H* = 36.867, FI ratio; *F* = 17.027, both *P* < 0.001). However, there was no difference among the AJP, CR and CM groups. The CR group showed a significant decrease in the Cobb angle compared with the other groups (*F* = 6.801, *P* < 0.001). There were no significant group differences in FMCSA and TMCSA (*H* = 0.087, *P* = 0.993, age; *F* = 1.905, *P* = 0.134).

**Table 2:** Comparison of the mean FCSA, FMCSA, TMCSA, FI ratio, and Cobb angle among four groups

	AJP group (n = 24)	CR group (n = 21)	CM group (n = 21)	HV group (n = 28)	*F/H* value	*P* value
FCSA (mm^2^)	86.65 (72.02, 122.72)	81.00 (62.85, 108.50)	96.80 (76.05, 137.80)	54.05 (46.77, 67.62) ^a^	36.867	< 0.001
FMCSA (mm^2^)	207.05 (167.37, 242.30)	191.30 (176.65, 243.45)	207.00 (184.50, 237.55)	201.05 (181.65, 259.90)	0.087	Ns
TMCSA (mm^2^)	314.09 ± 84.53	291.25 ± 77.70	314.50 ± 62.18	273.50 ± 61.95	1.905	Ns
FI ratio (%)	31.14 ± 7.44	28.92 ± 6.05	33.88 ± 7.33	21.26 ± 5.67 ^a^	17.027	< 0.001
Cobb angle (°)	12.29 ± 9.35	5.00 ± 7.84 ^b^	17.95 ± 12.62	14.04 ± 8.23	6.801	< 0.001

^*a*^*Compared with HV group*; ^*b*^*Compared with CR group*

In Table 3, the male subjects showed a significant increase in FCSA, FMCSA and TMCSA compared with the female subjects, while no significant difference was found in the FI ratio (*F*= -1.359, *P* = 0.179). In addition, there were no significant group differences in age (Table 4).

**Table 3:** Comparison of the mean FCSA, FMCSA, TMCSA and FI ratio by sex in patients with cervical spondylosis

	Male (n = 35)	Female (n = 31)	*T* value	*P* value
FCSA (mm^2^)	112.28 ± 33.07	78.32 ± 21.85	-4.971	< 0.001
FMCSA (mm^2^)	236.01 ± 61.76	181.94 ± 29.50	-4.618	< 0.001
TMCSA (mm^2^)	348.30 ± 77.10	260.27 ± 36.75	-6.025	< 0.001
FI ratio (%)	32.43 ± 7.57	30.04 ± 6.58	-1.359	Ns

**Table 4:** Comparison of mean FCSA, FMCSA, TMCSA and FI ratio dependent on age in patients with cervical spondylosis

	35 to 45 (n = 13)	46 to 55 (n = 26)	56 to 69 (n = 27)	*F* value	*P* value
FCSA (mm^2^)	95.49 ± 35.03	94.46 ± 30.19	98.54 ± 35.48	0.104	Ns
FMCSA (mm^2^)	234.73 ± 77.21	210.78 ± 54.10	198.84 ± 42.90	1.849	Ns
TMCSA (mm^2^)	330.23 ± 105.26	305.24 ± 68.85	297.39 ± 64.74	0.838	Ns
FI ratio(%)	29.00 ± 6.57	31.03 ± 7.03	32.68 ± 7.50	1.195	Ns

As shown in Table 5, in the cervical spondylosis group, the FCSA, and TMCSA were significantly correlated with Cobb angle (FCSA; r_s_= 0.336, *P* = 0.006, TMCSA; r_s_= 0.319, *P* = 0.009). A higher FI ratio was associated with poorer sagittal balance and older age, which was found to be inversely correlated with Cobb angle (r_s_= -0.285, *P* = 0.020) and positively correlated with age (r_s_= 0.261, *P* = 0.034). In the HV group, FMCSA was inversely correlated with the age (r_s_= -0.400, *P* = 0.035), while the FI ratio had a positive correlation with age (r_s_ = -0.423, *P* = 0.025).

**Table 5:** Correlation coefficients of FCSA, FMCSA, TMCSA, FI ratio and age, BMI and Cobb angle

	Groups	Age	BMI	Cobb angle
FCSA (mm^2^)	Cervical spondylosis group	0.092 (0.463)	0.124 (0.321)	0.336 (0.006) ^*^
	HV group	0.134 (0.497)	-0.171 (0.385)	0.304 (0.116)
FMCSA (mm^2^)	Cervical spondylosis group	-0.165 (0.185)	0.103 (0.409)	0.208 (0.094)
	HV group	-0.400 (0.035) ^*^	-0.009 (0.962)	0.187 (0.342)
TMCSA (mm^2^)	Cervical spondylosis group	-0.045 (0.720)	0.138 (0.268)	0.319 (0.009) ^*^
	HV group	-0.309 (0.110)	-0.073 (0.712)	0.285 (0.141)
FI ratio (%)	Cervical spondylosis group	0.261(0.034) ^*^	-0.064 (0.609)	-0.285 (0.020) ^*^
	HV group	0.423 (0.025) ^*^	-0.191 (0.331)	0.211 (0.280)

*P < 0.05

## Discussion

In this study, we demonstrate that a more severe degree of FI in the multifidus musculature and sagittal imbalance are found in patients with cervical spondylosis than in healthy controls. FI in the cervical multifidus muscle of the AJP syndrome were similar to those reported in persons with whiplash-associated disorders [4, 23, 24], as well as in patients with mechanical neck pain [5]. AJP syndrome of cervical spondylosis is derived from the pain and stiffness of zygapophyseal joint, which induce restricted movement and muscular weakness in cervical spine [25]. Atrophy and weakness of cervical multifidus muscles would lead to the increased FI. Kim et al. [6] reported that there was significant atrophy and FI of the cervical extensor in a patient complaining of chronic neck pain. The measurement of multifidus FI is still in its infancy in CR syndrome and CM syndrome. In this study, we demonstrate that the FI is significantly higher among patients with CR syndrome and CM syndrome than among age-matched controls. CR syndrome indicates that the compression or irritation of nerve roots in the neck region, which can cause pain, tingling, weakness and motor dysfunction in upper extremities [26]. This often causes the patient to present with a stiff neck and a decrease in range of motion, leading to the secondary musculoskeletal problems for reduction in muscle strength of the cervical spine musculature [27]. Additionally, CR syndrome is a broad disorder with peak prominence between the ages of 40 to 50 [28]. It may be a combination of nerve root dysfunction causing FI along with the muscular disuse and aging. Likewise, FI is known to occur following the denervation of skeletal muscle [12], including muscle denervation due to CM syndrome [29]. This is hypothesized to occur as spinal compression limits efferent output to these muscle motor units, leading to the disuse and atrophy in cervical extensor muscles [20]. Hence, FI is also considered to be an important concomitant phenomeon of cervical spondylosis.

We also investigated the relationship between FI and static postural stability. Changes in muscle composition such as increased FI of the deep, not superficial, extensors probably have direct implications on the function of the cervical spine muscles and mechanics. Deep neck extensors provide physical support to the spine vertebral column and play an important role in postural biomechanics, proprioception, and fine motor control [30, 31]. Previous clinical studies reported that patients with CR syndrome often present with cervical sagittal vertical misalignment resulting in forwards head posture and a reduced Cobb angle [32, 33], which correlates well with our findings. In this study, a higher FI ratio was found to be inversely correlated with the Cobb angle. The more severe the FI is, the worse the sagittal imbalance. Passias et al. [34] also reported that atrophic changes in multifidus musculature are associated with worsening cervical deformity. Despite the interdependence of cervical alignment and extensor musculature function, few studies have quantitatively described this relationship. In this study, we found that TMSCA is significantly higher among patients with cervical spondylosis than among healthy controls. One could speculate that the multifidus musculature of patients with cervical spondylosis has a compensatory hypertrophy process due to the continuous increase in FI in the long-term process of degeneration. Changing the physical properties of cervical musculature can alter its function [35]. The multifidus musculature is a deep extensor muscle, and has been shown to play a critical role in the biomechanical stability of the spine [36]. Accordingly, dysfunction could lead to pain and microinstability. A comprehensive assessment of patients’ muscle function is necessary during the prevention and treatment of cervical spondylosis. Developing customized rehabilitation training plans for cervical paravertebral muscle function can enhance muscle strength, reduce FI, and improve paravertebral muscle function, thereby delaying muscle degeneration.

Additionally, we evaluated our analysis for demographic data such as age, sex, and BMI in assessing the relationship with FI, since composition of the musculature may also be influenced by physical characteristics. The results of our study also reported that as age increased, there was a gradual decrease in FMCSA and a corresponding gradual increase in FI of the cervical multifidus musculature. Regarding the gender comparison, no significant difference in FI was found. There is not quite consistent with recent research. Chua et al. [37] reported that female patients may develop more severe and functionally significant multifidus atrophy, since the study was to determine if gender differences of multifidus FI in patients with lumbar spinal stenosis. This study indicated that increased muscle FI is a common tendency of musculoskeletal degeneration.

This study was limited by several factors. First, this is a retrospective, single-centre study. The possible selection bias is an inherent limitation. Thus, our study could be improved with a prospective study of a larger sample size, a longer follow-up period and more implant systems in the future. Second, the cervical multifidus musculature was only measured by MRI. Theoretically, the measurement of multiple muscles can provide a more comprehensive understanding. However, in deep cervical muscles, it is suggested that the cervical multifidus muscle has significantly larger amounts of FI than other cervical muscles [3]. The structural sensitivity of intramyocellular lipids differs on the basis of the fibre type, and type I fibres tend to be more easily altered than type II fibres [38, 39] since the cervical multifidus musculature has a high percentage of type I fibres.

## Conclusion

A more severe degree of FI in the multifidus musculature and sagittal imbalance are found in patients with cervical spondylosis than in healthy subjects. These two imaging features are considered to be important concomitant phenomena of cervical spondylosis, and the more severe FI is, the worse the sagittal imbalance. FI of cervical multifidus musculature presence on MRI is a physiologic degeneration and likely thought of as a potential pathology marker of cervical spondylosis, as it will be able to better refine decision-making prior to surgery and illustrate the role of therapy and rehabilitation in patients with cervical spondylosis.

## Data Availability

To avoid privacy being damaged from the research participants, the data will not be disclosed. Requests for data without being shown in this manuscript can be made to the corresponding author.

## Abbreviations

FI: Fat infiltration
HV: Healthy volunteers
CR: Cervical radiculopathy
DM: Degenerative myelopathy
AJP: Axial joint pain
FCSA: Fat cross-sectional area
FMCSA: Functional muscle cross-sectional area
TMCSA: Total muscle cross-sectional area
T2WI: T2-weighted images
